# Performance of small cluster surveys and the clustered LQAS design to estimate local-level vaccination coverage in Mali

**DOI:** 10.1186/1742-7622-9-6

**Published:** 2012-10-12

**Authors:** Andrea Minetti, Margarita Riera-Montes, Fabienne Nackers, Thomas Roederer, Marie Hortense Koudika, Johanne Sekkenes, Aurore Taconet, Florence Fermon, Albouhary Touré, Rebecca F Grais, Francesco Checchi

**Affiliations:** 1 Epicentre, Paris, France; 2 Médecins Sans Frontières, Bamako, Mali; 3 Médecins Sans Frontières, Paris, France; 4 National Centre for Immunization, Ministry of Health, Bamako, Mali

**Keywords:** Vaccination coverage, Mali, Meningitis, Lot quality assurance sampling, LQAS, Cluster sampling, Survey

## Abstract

**Background:**

Estimation of vaccination coverage at the local level is essential to identify communities that may require additional support. Cluster surveys can be used in resource-poor settings, when population figures are inaccurate. To be feasible, cluster samples need to be small, without losing robustness of results. The clustered LQAS (CLQAS) approach has been proposed as an alternative, as smaller sample sizes are required.

**Methods:**

We explored (i) the efficiency of cluster surveys of decreasing sample size through bootstrapping analysis and (ii) the performance of CLQAS under three alternative sampling plans to classify local VC, using data from a survey carried out in Mali after mass vaccination against meningococcal meningitis group A.

**Results:**

VC estimates provided by a 10 × 15 cluster survey design were reasonably robust. We used them to classify health areas in three categories and guide mop-up activities: i) health areas not requiring supplemental activities; ii) health areas requiring additional vaccination; iii) health areas requiring further evaluation. As sample size decreased (from 10 × 15 to 10 × 3), standard error of VC and ICC estimates were increasingly unstable. Results of CLQAS simulations were not accurate for most health areas, with an overall risk of misclassification greater than 0.25 in one health area out of three. It was greater than 0.50 in one health area out of two under two of the three sampling plans.

**Conclusions:**

Small sample cluster surveys (10 × 15) are acceptably robust for classification of VC at local level. We do not recommend the CLQAS method as currently formulated for evaluating vaccination programmes.

## Introduction

Vaccination coverage (VC) estimates are essential to monitor the performance of immunisation programmes and take action to improve them. In resource-poor settings, administrative estimates of VC, reached by dividing the number of people vaccinated by the population in the target age group, are often biased due to inaccurate population figures and pressure on programmes to report favourable indicators. Sample surveys are thus frequently employed to establish more accurate estimates.

A specific challenge in these settings is estimation of VC at the local level (e.g. district, sub-district or health catchment area), so as to identify communities that may require additional support (e.g. supplementary campaigns, strengthening of routine vaccination) and allocate limited resources efficiently. To do this, two survey methods recommended by the World Health Organization are available: cluster surveys and lot quality assurance sampling (LQAS) [1].

Cluster surveys feature simple designs that do not require accurate population figures or household sampling frames [2]. However, cluster samples cannot be used to make inferences for individual communities within the sampling universe; therefore, for each community of interest, one independent cluster sample needs to be selected. Typical sample sizes for such cluster surveys are on the order of 30 clusters x 7 individuals [3]. Theoretically even smaller samples may be chosen, but there is insufficient evidence on whether the resulting estimates are likely to be robust, i.e. whether both the point estimate and the estimated standard error (SE) remain acceptably stable as sample size decreases [4].

LQAS has been promoted as a faster and cheaper alternative to cluster surveys for monitoring various public health interventions [5], though it could be potentially misused due to erroneous statistical assumptions [6]. In this approach, a random sample N of individuals (or other basic sampling units, depending on the indicator being monitored) is selected within each community, or lot. LQAS yields a binary classification decision: in the case of vaccination, the lot is “rejected” (i.e. judged to require supplementary activities) if the number of unvaccinated individuals within the sample exceeds a decision threshold d, and “accepted” otherwise. Various sampling plans consisting of a given N and d can be used. However, in practice where both time and resources are often limited, one needs to specify a lower threshold VC (LT), i.e. the minimum acceptable VC below which supplementary interventions (e.g. re-vaccination) must take place; and an upper threshold VC (UT), usually fixed at the target VC. Each sampling plan features a probability α that the survey will yield an acceptance decision when in fact the lot has a VC < LT (this is known as the “consumer” risk, as it deprives beneficiaries of the intervention they need); and a probability β that the lot will be rejected when in fact VC exceeds the UT (this constitutes the “provider” risk of expending resources needlessly). Minimising the consumer risk is the main criterion for selecting a sampling plan. Minimising the provider risk is also important, but in many situations a relatively high provider risk is tolerated so as to ensure that the resulting sample size still makes LQAS more advantageous than a standard survey. The combination of a large grey zone between LT and UT (a result of the sampling plan) and a high proportion of communities falling within this grey zone (a phenomenon independent of the sampling plan, but merely reflecting how the variable is distributed in the population) however results in a high classification error [7].

The theoretical advantage of LQAS is that it yields the desired information with much smaller sample sizes than cluster surveys. However, the often-overlooked requirement for a fully random sample poses a serious challenge in resource-poor settings, since updated lists of households are often unavailable, and since random sampling will usually require travel to an unfeasibly large number of sites within the community.

To overcome this problem, Pezzoli et al. and Greenland et al. [8-10] have recently put forward a more field-friendly “clustered LQAS” (CLQAS) approach, whereby the lot sample is divided into clusters, as in any multi-stage cluster sample. The critical assumption behind this approach is that, within any given lot (e.g. a district), the true VC levels in the different individual primary sampling units (e.g. villages), among which one would randomly select clusters, always give rise to a binomial distribution, with the mean of this distribution equal to the overall VC of the lot, and the standard deviation equal to or less than an a priori assumed level. The authors propose various sampling plans (e.g. 5 clusters of 10 individuals) that, for assumed standard deviations ≤ 0.05 or ≤ 0.10 and typical LT and UT thresholds of interest, yield reasonably low α and β probabilities.

As estimates of local vaccination coverage are used to orient subsequent catch-up vaccination activities, the choice of appropriate survey methodology is essential. The CLQAS approach has been used in different settings including Nigeria and Cameroon [8,9]; however, the accuracy of classifications generated by this design and implications of this accuracy for operational decisions have not been sufficiently documented [11]. Using data from a vaccination coverage survey carried out in Mali in January 2011, we aimed to evaluate the performance of CLQAS in a typical field setting. We also explored whether classical surveys using smaller samples than currently recommended provide results that, although less precise, are still statistically stable and useful for operational decision-making, and could thus constitute an alternative to CLQAS.

## Methods

### Cluster survey

A new, single-dose conjugate vaccine against meningococcal meningitis group A (MenAfriVac®) that confers long-term immunity has recently completed development [12]. Three countries were selected for the initial introduction of the vaccine: Burkina Faso, Mali and Niger. In these countries, mass campaigns were carried out with a target of ≥ 90% VC in the age group 1 to 29 years. Médecins Sans Frontières (MSF) supported the vaccination campaign in three districts of the Koulikoro region in Mali during December 2010. We carried out a VC survey in one of these districts (Kati). The objectives of the survey were to estimate VC for the district as a whole and to identify health areas (*aires de santé*) with VC < 80%, thus eligible for catch-up activities.

The district of Kati is divided into 41 health areas. We therefore did a stratified multi-stage cluster survey, with each health area constituting a stratum. All individuals aged 1 to 29 years at the time of vaccination and living in Kati were eligible. The basic sampling unit was the household (defined as a group of people living under the responsibility of a single head of household and who eat and sleep together), with all eligible individuals in a household included in the survey. We targeted a sample of 123 individuals per health area, sufficient to estimate a VC of 80% with precision ± 10% and a design effect (DEFF) of 2. Assuming 3 persons in the eligible age group per household, and a 10% household non-participation rate, we required 46 households to achieve this sample. We thus sampled 10 clusters of 5 households per health area. Over all 41 health areas, this yielded a target sample of 410 clusters containing 6 150 people, sufficient to estimate a VC of 80% with precision ± 2% and a DEFF of 4.

Kati district surrounds the city of Bamako. Ten of the health areas feature a densely populated peri-urban layout, while the remainders comprise rural areas with lower population density. In peri-urban areas, clusters were allocated spatially. Accordingly, we mapped the contours of each health area using global positioning system (GPS) devices, and selected the starting point of each cluster randomly from the intersection points of a grid overlaid on the map, as in Grais et al. [13]. The first house (or compound) visited in the cluster was that closest to the selected intersection point. If more than one household lived in the house or compound, we selected one of these at random. The second house visited was the second on the left; and so forth until 5 households containing at least one person in the eligible age group had been visited.

In rural areas, cluster starting points were selected using probability proportional to size sampling, based on a sampling frame of villages and their administrative population estimates. From the geographic centre of the village containing the cluster, interviewers numbered all houses up to the village edge along a random direction and chose one at random as the starting house in the cluster (this is known as the Expanded Programme on Immunization or spin-the-pen method [14]). Further houses were selected as above.

In each household, after gathering verbal consent, investigators interviewed all eligible individuals (or their caregivers for children) in the local language using a standardised questionnaire. Individuals were considered vaccinated if they provided verbal confirmation or based on their immunisation card, when available. VC estimates and DEFF were used to classify health areas in categories to guide mop-up activities.

The study was implemented in collaboration with the Ministry of Health after obtaining permission to carry out the survey. The survey was conducted by 18 teams of two persons after three days of training, including a pilot field test. Data collection took place from 15 to 24 January 2011. Data were entered in EpiData 3.1 (The EpiData Association, Odense, Denmark) and analysed using R software [15]. Stratum-specific VC estimates were weighted for selection of single households within houses and for unequal cluster sizes, while the estimate for the entire district was also weighted for unequal stratum population sizes.

### Exploring the performance of small cluster survey designs and CLQAS

Using the full survey database, we firstly explored the stability of health area-specific estimates of VC obtained through the 10 clusters x 15 individuals cluster design used in Mali, or smaller sample sizes obtained by further reducing the number of individuals per cluster. Such small cluster surveys could provide a reasonable alternative to (C)LQAS without necessitating the definition of an upper and lower threshold. Next, we performed a simulation of alternative CLQAS designs in order to explore their performance for all health areas including those falling in the interval between the UT and LT (grey zone). Although the choice of the grey zone should balance feasibility with classification accuracy, a large number of health areas falling within the grey zone would all but undo a CLQAS survey’s operational usefulness.

First, using the survey database, we created samples of decreasing size for each health area, from 10 clusters x 15 individuals to 10 × 3, by eliminating observations from the database, starting from the last person interviewed in each cluster.

We then investigated the stability of the VC point estimate, SE and intra-cluster correlation coefficient (ICC) of VC in these progressively smaller samples, as a measure of their statistical robustness. To do this, for each health area and sampling design (i.e. from 10 × 15 to 10 × 3), we drew 10 000 bootstrap samples from the original data, using a bootstrap sampling procedure recommended for cluster survey data [16], which consists of sampling entire clusters with replacement, without further re-sampling of observations within clusters.

We analysed the distribution of bootstrap samples to compute the precision of VC, SE and ICC, as in Efron and Tibshirani [17]. Absolute precision was computed as (97.5% percentile of distribution - 2.5% percentile)/2.

Second, using the full survey database, we simulated a CLQAS design consisting of 10 clusters of 5 individuals per health area (lot), i.e. the same sample size recommended by Pezzoli et al. [10], but with double the number of clusters and half the number of individuals per cluster, i.e. tending towards lower DEFF and thus greater precision. Accordingly, for each health area we drew 10 000 independent random samples of 5 individuals within each of the 10 clusters, with the constraint that sampled individuals must not belong to the same household. We computed the number of unvaccinated individuals arising from each of these replicate samples, and applied alternative decision values (d) and LT, UT thresholds suggested by Pezzoli et al. [9,10] to accept or reject the area. See Table 1 for the theoretical error risks associated with these sampling plans, based on Pezzoli et al.’s assumption of a binomial distribution of lot VC, with SE ≤ 0.10.

**Table 1:** CLQAS sampling plans included in the evaluation

**Sampling plan**	**Lot sample size (N)**	**Lower VC threshold (LT)**	**Upper VC threshold (UT)**	**Decision threshold (d)**	**Consumer error risk (α)†**	**Provider error risk (β)‡**
**1**	50 (10 × 5)	85%	95%	3	10%	30%
**2**	50 (10 × 5)	80%	95%	4	5%	19%
**3**	50 (10 × 5)	75%	90%	7	8%	20%

These sampling plans assume that vaccination coverage in the 10 clusters is distributed according to a binomial distribution centred at the point estimate for the entire lot, and with SD ≤ 0.10. If the number of unvaccinated individuals > d, the lot is rejected.

†Probability of classifying the lot as “acceptable” when in fact VC < LT. ‡ Probability of classifying the lot as “unacceptable” when in fact VC ≥ UT. Probabilities for plan A are taken from Table one of Pezzoli et al. [9], and for B and C from Tables two and three in Pezzoli et al., [10] respectively. Note that all calculations in these publications refer to a 5 × 10 (i.e. theoretically less precise) plan.

For each health area and sampling plan, we computed the proportion of simulations leading to rejection of the health area (i.e. re-vaccination). We also compared the CLQAS classification with that provided by the point estimate of the cluster survey to compute the frequency of “correct” classification including health areas in the grey zone.

## Results

### Cluster survey

In total, 2188 households were visited in the 41 health areas of the district of Kati. Ten refused to participate (0.5%) and 117 were absent after two visits (5.3%). We thus interviewed a total of 2061 households, of which 2050 contained at least one individual from the target age group (1-29 years old). A total of 21 367 people were included in the survey, of which 73% (n = 15 668; mean of 7.6 per household) were in the target age group for the vaccination campaign, with a male to female ratio of 0.9.

Among the target age group, VC (by immunization card or verbal confirmation) was estimated at 88.4% (95%CI 85.7-90.6). Table 2 shows VC by age group and sex. Male adults (15-29 years old) had the lowest VC. The main reported reasons for non-vaccination were absence during vaccination activities (29.5%), believing not to be part of the target population (15.0%) and lack of information about the campaign (9.6%).

**Table 2:** District-wide estimates of vaccination coverage, by age group and sex

**Age group**	**Male**		**Female**		**Total**	
	**N**	**% (95%CI)**	**N**	**% (95%CI)**	**N**	**% (95%CI)**
<1 year	252	3.8 (1.0-12.7)	235	11.5 (5.0-24.3)	489	10.9 (5.4-20.8)
1-4 years	1682	92.5 (88.8-95.0)	1767	91.0 (87.7-93.4)	3455	92.0 (89.5-93.9)
5-14 years	3489	95.8 (94.0-97.1)	3589	94.4 (91.4-96.4)	7087	95.1 (92.7-96.7)
15-29 years	2046	70.0 (66.0-73.8)	3070	81.2 (76.5-85.2)	5126	77.4 (74.0-80.4)
>29 years	2713	12.7 (10.8-14.8)	2481	20.3 (18.6-22.1)	5210	16.3 (15.0-17.6)
Total	10,182	65.3 (63.5-66.9)	11,142	71.8 (69.4-74.1)	21,367	68.7 (66.7-70.6)

**Figure 1:**
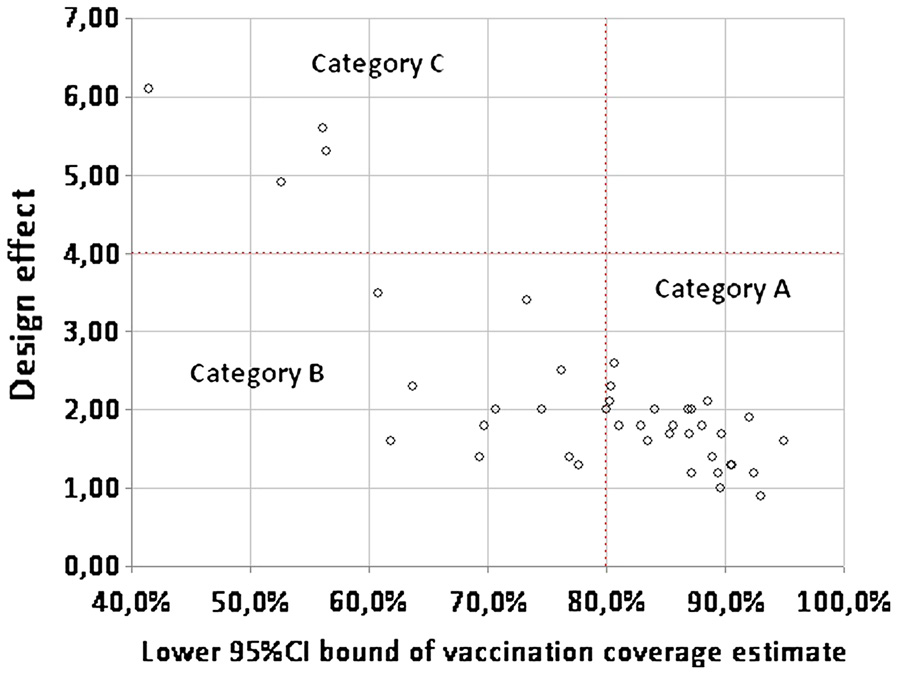
Classification of health areas (n=41) according to vaccination coverage estimates (95%CI lower bound) and design effect in Kati district.

VC among the target age group was also estimated for each health area. Table 3 shows the 41 health areas ranked by descending VC point estimate; the standard deviation of VC across the 10 clusters in the health area; the value of the design effect (DEFF) and intra-cluster correlation coefficient (ICC). These values were used to re-classify health areas in three categories to guide mop-up activities: category A included health areas where the lower bound of the 95%CI of the VC estimate was above 80%; category B included health areas where the lower bound of the 95%CI of the VC estimate was below 80% and therefore required additional vaccination activities; and category C, health areas where the DEFF was above 4.0 suggesting pockets of unvaccinated populations and thus requiring targeted catch-up vaccination activities. See the Discussion for limitations of this approach. Overall, 26 health areas were “accepted” and not requiring supplemental activities (cat. A); 11 health areas were “rejected” as requiring additional vaccination activities (cat. B); and 4 health areas were also “rejected” as requiring targeted catch-up vaccination (cat. C) (Figure 1). Cluster-level summaries for each health area are provided in Additional File 1 to facilitate for further simulation work based on this dataset.

**Table 3:** Estimates of vaccination coverage by health area

**Health area**	**VC point estimate (%)**	**95% confidence interval**	**Standard deviation of VC**	**Design effect**	**Intra-cluster correlation coefficient**	Classification for decision*
Neguela	98.3	95.0-99.4	0.030	1.6	0.014	A
Nana-Kenieba	96.7	92.0-98.7	0.048	1.9	0.018	A
Sandama	95.7	92.4-97.6	0.040	1.2	0.005	A
Kati Coro	95.6	89.7-98.2	0.062	1.7	0.021	A
Diago	95.4	93.0-97.0	0.031	0.9	−0.001	A
Kalifabougou	94.9	90.6-97.3	0.051	1.3	0.007	A
Dombila	94.4	88.5-97.4	0.067	2.1	0.024	A
Niouma-Makana	94.4	86.9-97.7	0.079	2.0	0.029	A
Faladie	94.3	90.5-96.6	0.048	1.3	0.008	A
Tanima	93.7	87.0-97.1	0.076	1.7	0.021	A
Falani	93.5	88.9-96.3	0.058	1.4	0.007	A
N'Gouraba	93.5	88.1-96.5	0.065	1.8	0.019	A
Yelekebougou	93.5	89.4-96.1	0.053	1.2	0.006	A
Kati Sananfara	93.2	85.6-96.9	0.085	1.8	0.025	A
Doubabougou	93.1	89.6-95.5	0.047	1.0	−0.001	A
Djoliba	92.8	87.2-96.0	0.068	2.0	0.022	A
Kanadjiguila	91.7	80.3-96.8	0.123	2.1	0.034	A
Sonikegny	91.5	87.2-94.4	0.057	1.2	0.007	A
Siby	91.0	84.1-95.1	0.086	2.0	0.022	A
Kabalabougou	90.9	85.3-94.5	0.072	1.7	0.022	A
Dogodouma	89.8	83.5-93.8	0.150	1.6	0.024	A
Sanankoroba	89.8	76.2-96.0	0.082	2.5	0.050	B
Siracoro Meguetana	89.5	80.4-94.7	0.111	2.3	0.033	A
Safo	89.3	81.1-94.2	0.102	1.8	0.024	A
Bancoumana	88.7	80.7-93.6	0.102	2.6	0.033	A
Moutougoula	88.6	82.9-92.5	0.077	1.8	0.018	A
Malibougou	87.6	73.3-94.8	0.165	3.4	0.059	B
Baguineda	87.3	80.0-92.2	0.097	2.0	0.027	A
Diedougou Torodo	83.6	56.4-95.2	0.085	5.3	0.091	C
Sangarebougou	83.6	77.7-88.3	0.303	1.3	0.009	B
Farada	83.2	74.6-89.3	0.118	2.0	0.025	B
Kalabancoro	82.9	76.9-87.6	0.086	1.4	0.012	B
Dialakorodji	82.1	56.1-94.3	0.303	5.6	0.170	C
Kalabancoro Koulouba	80.8	70.7-88.0	0.139	2.0	0.038	B
Kalabancoro Adeken	79.8	69.7-87.1	0.140	1.8	0.035	B
Daban	79.1	52.6-92.8	0.327	4.9	0.091	C
Kati Koko	77.9	60.8-88.9	0.229	3.5	0.084	B
N'Gabacoro-Droit	76.3	69.3-82.2	0.104	1.4	0.015	B
Dio-Gare	76.1	41.5-93.5	0.441	6.1	0.134	C
Moribabougou	73.6	63.7-81.6	0.146	2.3	0.030	B
Kalabancoro Heramakono	71.7	61.8-79.9	0.148	1.6	0.027	B

*A= “accepted”; B= “rejected”, requiring overall additional vaccination activities; C= “rejected”, requiring targeted catch-up vaccination activities.

**Table 4:** Results of the CLQAS simulation (10 000 runs), for three alternative sampling plans

			**Sampling plan 1**			**Sampling plan 2**			**Sampling plan 3**		
			**(LT = 85%, d = 3)**			**(LT = 80%, d = 4)**			**(LT = 75%, d = 7)**		
**Health area**	Survey VC estimate (%)	CLQAS median number unvaccinated individuals (95% percentile)	VC region	Frequency of "reject" classification	Frequency of correct classification†	VC region	Frequency of "reject" classification	Frequency of correct classification†	VC region	Frequency of "reject" classification	Frequency of correct classification†
Neguela	98.3	1 (0–2)	>UT	0.002	0.999	>UT	0.000	1.000	>UT	0.000	1.000
Nana-Kenieba	96.7	3 (1–5)	>UT	0.235	0.765	>UT	0.067	0.933	>UT	0.000	1.000
Sandama	95.7	3 (1–6)	>UT	0.368	0.632*	>UT	0.157	0.843	>UT	0.001	0.999
Kati Coro	95.6	3 (1–6)	>UT	0.498	0.502*	>UT	0.239	0.761*	>UT	0.004	0.996
Diago	95.4	3 (0–6)	>UT	0.272	0.728	>UT	0.097	0.904	>UT	0.001	0.999
Kalifabougou	94.9	4 (1–7)	Grey	0.614		Grey	0.374		>UT	0.019	0.981
Dombila	94.4	3 (1–6)	Grey	0.428		Grey	0.201		>UT	0.004	0.996
Niouma-Makana	94.4	5 (2–8)	Grey	0.755		Grey	0.517		>UT	0.038	0.962
Faladie	94.3	3 (1–6)	Grey	0.442		Grey	0.202		>UT	0.003	0.998
Tanima	93.7	4 (1–7)	Grey	0.550		Grey	0.292		>UT	0.009	0.991
Falani	93.5	3 (0–6)	Grey	0.364		Grey	0.162		>UT	0.002	0.998
N'gouraba	93.5	3 (1–7)	Grey	0.436		Grey	0.216		>UT	0.007	0.993
Yelekebougou	93.5	4 (1–7)	Grey	0.573		Grey	0.343		>UT	0.018	0.982
Kati Sananfara	93.2	5 (3–8)	Grey	0.900		Grey	0.683		>UT	0.064	0.936
Doubabougou	93.1	3 (1–6)	Grey	0.400		Grey	0.194		>UT	0.005	0.995
Djoliba	92.8	4 (2–7)	Grey	0.697		Grey	0.417		>UT	0.018	0.982
Kanadjiguila	91.7	8 (5–11)	Grey	1.000		Grey	0.992		>UT	0.553	0.447*
Sonikegny	91.5	4 (1–7)	Grey	0.585		Grey	0.348		>UT	0.018	0.982
Siby	91.0	5 (2–9)	Grey	0.864		Grey	0.702		>UT	0.133	0.867
Kabalabougou	90.9	5 (2–8)	Grey	0.761		Grey	0.520		>UT	0.038	0.963
Dogodouma	89.8	6 (3–9)	Grey	0.947		Grey	0.799		Grey	0.108	
Sanankoroba	89.8	6 (3–9)	Grey	0.964		Grey	0.863		Grey	0.193	
Sirac. Meguetana	89.5	7 (3–11)	Grey	0.958		Grey	0.874		Grey	0.331	
Safo	89.3	6 (3–10)	Grey	0.957		Grey	0.847		Grey	0.235	
Bancoumana	88.7	6 (2–9)	Grey	0.888		Grey	0.725		Grey	0.150	
Moutougoula	88.6	6 (3–10)	Grey	0.912		Grey	0.772		Grey	0.178	
Malibougou	87.6	8 (4–12)	Grey	0.992		Grey	0.966		Grey	0.550	
Baguineda	87.3	8 (5–12)	Grey	0.999		Grey	0.988		Grey	0.640	
Diedoug. Torodo	83.6	9 (6–12)	<LT	1.000	1.000	Grey	1.000		Grey	0.804	
Sangarebougou	83.6	10 (6–14)	<LT	1.000	1.000	Grey	0.996		Grey	0.850	
Farada	83.2	9 (5–14)	<LT	0.997	0.997	Grey	0.982		Grey	0.736	
Kalabancoro	82.9	8 (4–12)	<LT	0.983	0.983	Grey	0.947		Grey	0.546	
Dialakorodji	82.1	7 (4–10)	<LT	0.997	0.997	Grey	0.962		Grey	0.314	
Kalab. Koulouba	80.8	10 (6–14)	<LT	0.999	0.999	Grey	0.996		Grey	0.866	
Kalab. Adeken	79.8	12 (8–16)	<LT	1.000	1.000	<LT	1.000	1.000	Grey	0.985	
Daban	79.1	10 (7–13)	<LT	1.000	1.000	<LT	1.000	1.000	Grey	0.937	
Kati Koko	77.9	8 (5–12)	<LT	0.999	0.999	<LT	0.987	0.987	Grey	0.673	
N'gabacoro-Droit	76.3	10 (6–14)	<LT	1.000	1.000	<LT	0.997	0.997	Grey	0.877	
Dio-Gare	76.1	10 (7–13)	<LT	1.000	1.000	<LT	1.000	1.000	Grey	0.955	
Moribabougou	73.6	14 (10–18)	<LT	1.000	1.000	<LT	1.000	1.000	<LT	0.998	0.998
Kalab. Heramak.	71.7	16 (13–20)	<LT	1.000	1.000	<LT	1.000	1.000	<LT	1.000	1.000

†Using the VC point estimate as a reference.

*Error higher than expected (>β).

### Performance of small cluster samples and CLQAS

**Figure 2:**
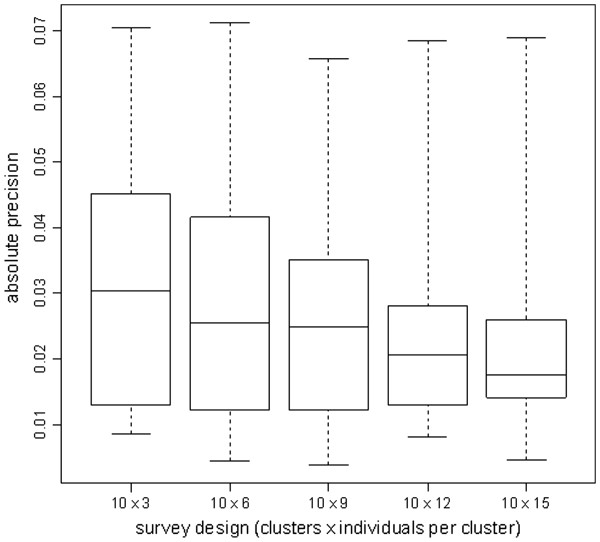
**Absolute precision of estimates of the standard error of VC, for different survey designs. **Box plots indicate the median and inter-quartile range of the median absolute precision values from 10 000 bootstrap replicates of each of the 41 stratum surveys. Whiskers denote the range.

**Figure 3:**
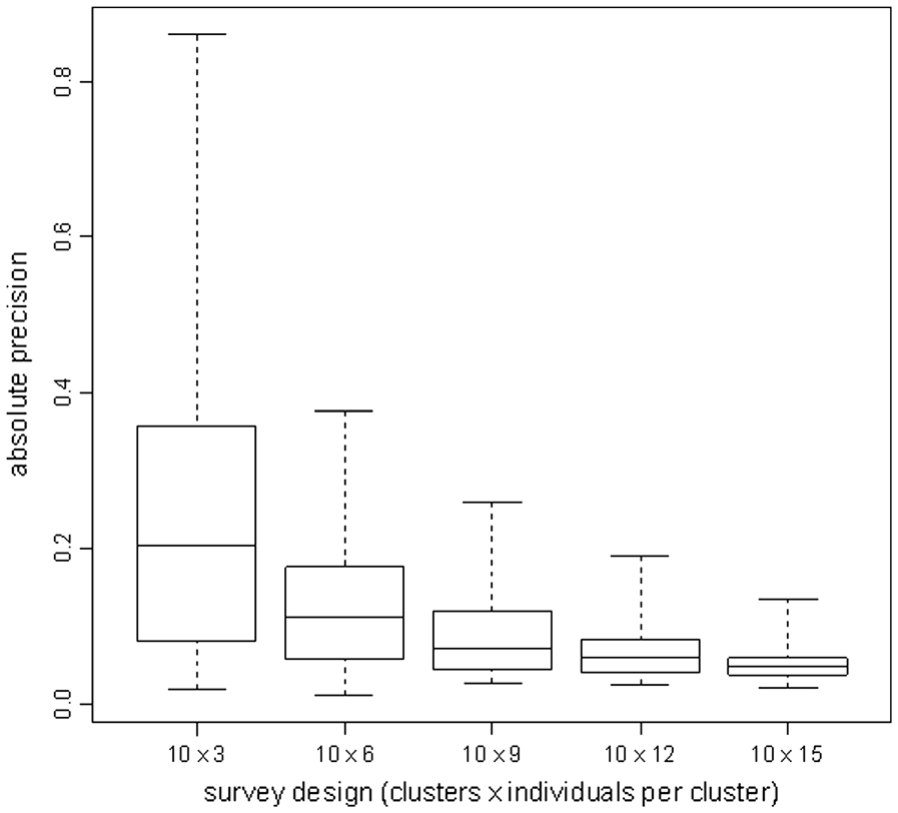
**Absolute precision of estimates of the intra-cluster correlation coefficient of VC, for different survey designs. **Box plots indicate the median and inter-quartile range of the median absolute precision values from 10 000 bootstrap replicates of each of the 41 stratum surveys. Whiskers denote the range.

Exploration of alternative sampling plans for the small cluster design suggests that, as sample size decreased from 10 × 15 to 10 × 3 individuals, the stability (absolute precision) of the standard error of VC decreased from a median of ±0.017 to ±0.030 across all 41 strata (Figure 2). ICC was markedly unstable at low sample sizes (Figure 3), ranging from a median absolute precision of ±0.047 (10 × 15 design) to a median precision of ±0.204 (10 × 3 design).

As expected, for the CLQAS, the proportion of simulations leading to rejection of each health area (i.e. re-vaccination) varied considerably and was dependent on the distribution of the number of unvaccinated individuals resulting from the simulation runs and on the sampling plan (Table 4).

**Figure 4:**
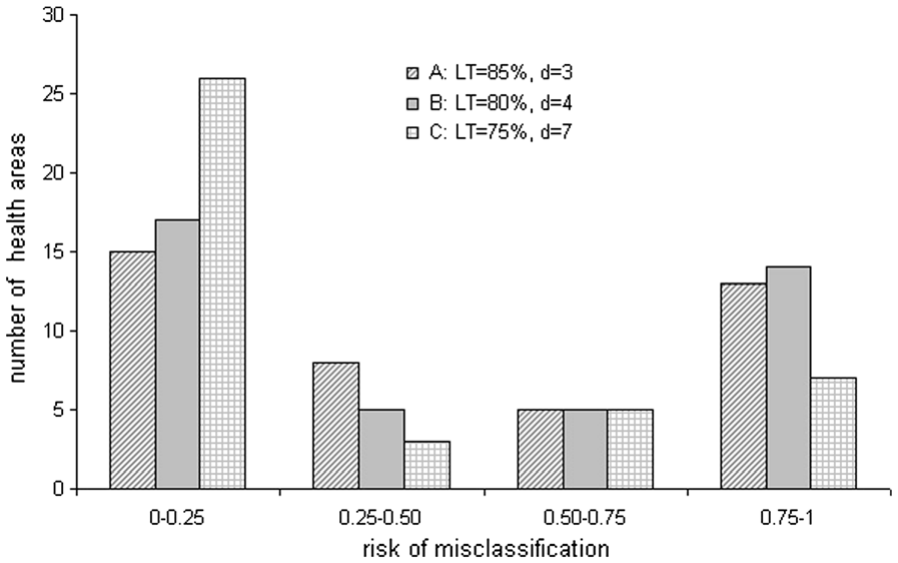
Distribution of CLQAS misclassification risk, for three sampling plans, among all health areas.

When looking at health areas with a VC > UT, the CLQAS wrongly rejected, with a probability β greater than expected, two areas for sampling plan 1, one area for sampling plan 2 and one area for sampling plan 3. When looking at health areas with VC < LT, none was wrongly accepted and all probabilities α were below the expected maximum. However, when looking at health areas that were rejected with a VC > UT or accepted with VC < LT and also including health areas in the grey zone, at least one third of health areas had a risk of misclassification ≥ 0.25, irrespective of sampling plan. Under plans A and B, about half of lots had a risk greater than 0.50 of being misclassified (Figure 4). Nearly all misclassification error was on the provider side, thereby potentially resulting in unwarranted re-vaccination of health areas with good VC.

## Discussion

### Cluster survey

VC for the entire district of Kati was high and close to the target of ≥ 90%. Data collected for each stratum of the cluster survey allowed us to interpret results for each health area and classify areas accordingly into categories: health areas not requiring further corrective measures (VC ≥ 80%); health areas requiring additional vaccination activities (VC possibly under 80%); health areas without an acceptable precision of VC estimates (high degree of heterogeneity or DEFF over 4.0), indicating the existence of pockets of unvaccinated individuals. For these areas the recommendation was to carry out further investigations through local informants in order to identify specific communities with low VC to be targeted by catch up campaigns. The above classification system is also potentially flawed: confidence intervals, particularly for proportions, are known to have imperfect coverage (e.g. 95% intervals rarely contain the true value 95% of the time as expected) [18]; moreover, the DEFF cut-offs we used are arbitrary and, while they seemed useful in this setting, would have to be formally tested in a variety of other scenarios, with classification properties evaluated against a known gold standard. While we don’t suggest that our classification system should be adopted uncritically instead of CLQAS, we nonetheless believe that one need not to look to LQAS alone as a way to meaningfully use small survey data, and that both the confidence interval and the observed degree of clustering provide useful information for classification. In particular, we suggest that the estimated ICC value could be used in the future to refine classification as opposed to relying only on DEFF.

In order to provide information at the local (health area) level, we opted for small stratum surveys of 10 clusters, far lower than the recommended 30 clusters. Investigation of the statistical robustness of this design and increasingly smaller cluster samples suggested that, with fewer than 10 clusters x 15 individuals, standard error and ICC estimates were increasingly unstable. However, the 10 × 15 design appeared to provide reasonably robust estimates in most health areas: an absolute precision of ±0.015 to ±0.025 in the standard error of VC roughly means that, 95% of the time, confidence intervals returned by the 10 × 15 design in this setting would have been accurate within about ±3 to ±5%. Our analysis suggests that, for local *classification* of VC within the context of a larger survey aiming to estimate VC across a district or region, small stratum cluster samples of 10 clusters provide a reasonable balance between feasibility and statistical robustness. However, our findings do not support a 10 cluster design for accurate estimation of VC.

### Performance of the clustered LQAS design

When considering the aim of providing information for decision-making at local level, the overall recommended sample size for a CLQAS design in the district was three times smaller (N=2 050 assuming a 10 × 5 design) than the sample size needed for our cluster survey. Further improvements in efficiency would have resulted from early stoppage of LQAS surveys when the number of unvaccinated individuals exceeded d, even before completing the lot sample.

Our simulation based on real field data showed that, in this setting, the CLQAS design always classified health areas with VC < LT correctly, and mostly classified correctly health areas with VC ≥ UT - that is, it almost always achieved the classification accuracy specified by each of the sampling plans tested. Moreover, all misclassifications were of a conservative nature, i.e. provider risk leading to unwarranted revaccination.

However, in practice, decision makers on the field need to adopt a binary decision for each health area - that is, either to carry out supplementary vaccination activities or to treat the area as sufficiently vaccinated. This means that the classification reached by the CLQAS method for areas that in reality fall within the “grey zone” is also highly relevant for operations: areas that are “rejected” would go on to receive additional vaccination interventions. Our simulation shows that, in our Mali scenario, all three of the sampling plans tested leave a large proportion of health areas in the grey zone, where the risk of misclassification is very high. For many health areas, CLQAS appeared no better than flipping a coin. The consequence of this high risk of misclassification would mainly have been to allocate resources for catch up campaigns in areas where they were not needed. It seems plausible that the 5 × 10 design put forward by Pezzoli et al., while reaching the same sample size, would have performed even worse given the lower ratio of clusters to individuals. In operational terms, our results suggest that, in choosing to save resources upfront by reducing the cost of surveys through LQAS, vaccination programmes may in fact end up committing even greater resources down the line by having to carry out remedial vaccination in a far greater proportion of the community than in fact needed.

To a large extent, the above findings reflect a known limitation of LQAS: its specificity is high only if few of the lots fall within the grey zone [19]. However, additional inaccuracy in our results also arose from the violations of two key assumptions of CLQAS that are irrelevant if the traditional LQAS method featuring simple or systematic random sampling (SRS) is carried out. The first assumption is that the standard deviation of VC in any cluster within each lot does not exceed a given value (0.10 in the sampling plans we studied). This assumption has been shown to be violated for 25-50% of lots in applications of the CLQAS to date [8,9], meaning that variability of VC within the lot is in fact often greater than expected. In this study, 17/41 (41.4%) of health areas featured a standard deviation > 0.10 (Table 3), reinforcing the above findings. It should be noted, however, that standard deviation values presented in this study may or may not reflect the true standard deviations of VC in villages within each health area, since the cluster-level samples were collected through a sampling process (i.e. random walk) that is not designed to return a representative sample of the community within which the cluster falls.

**Figure 5:**
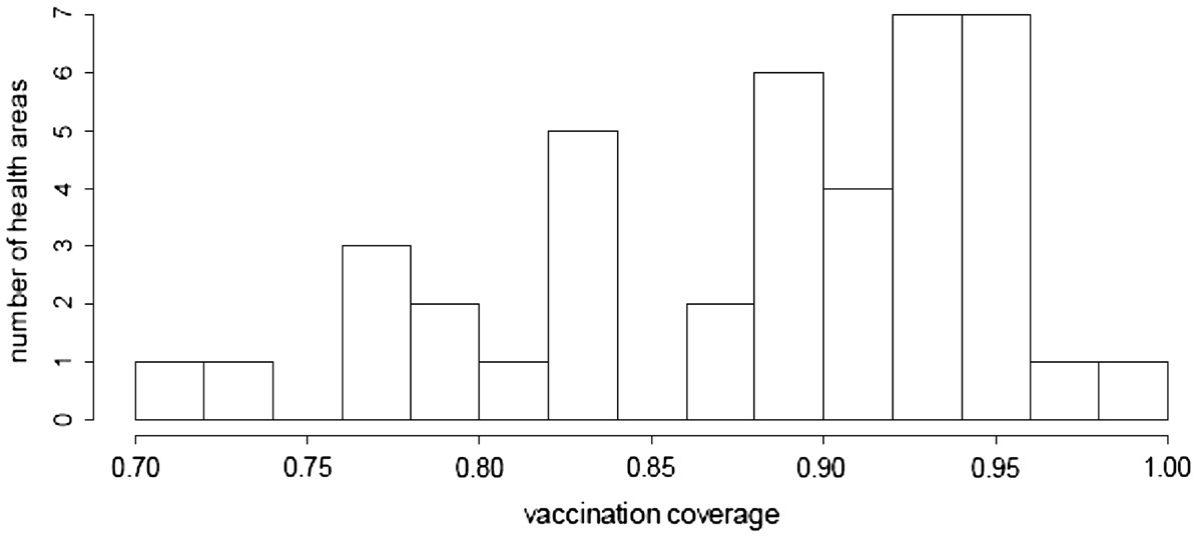
Distribution of vaccination coverage in Kati district (n = 41 health areas).

The second, more fundamental assumption is that VC within each lot follows a binomial distribution. In developing countries, multi-modal or over-dispersed (left- or right-skewed) distributions of VC are more likely given the known difficulties in accessing remote communities and the very uneven performance of local health services [20]. The distribution of VC across health areas in our district of intervention also suggests a pattern other than binomial, though for an administrative level higher than the one at which we evaluated the CLQAS (Figure 5).

If cluster sampling is to be used with LQAS, the method’s accuracy could be increased by reducing inter-cluster variability within lots. This can be done by either (i) increasing the number of clusters or (ii) working at a smaller geographic resolution (e.g. lots defined as sub-districts or villages, within which VC may be more homogeneous). Both solutions would however negate the main advantage of LQAS, as the resulting survey would be neither faster nor cheaper than a stratified cluster survey. Furthermore, in our study we already considered the smallest administrative division of relevance for vaccination programmes. A third solution would be to restrict the application of the method “to evaluate immunisation programmes that tend to perform well” or to scenarios “when the territory under study is somewhat homogeneous in terms of vaccination coverage” [8]. However, highly performing areas are rarely known in advance, and inter-cluster variability is difficult to predict; furthermore, such an approach would seem to negate the very purpose of carrying out VC studies.

### Study limitations

Limitations of the cluster survey, common to most VC surveys in developing countries, include potential misclassification bias due to the retrospective nature of data collection, and inability in many cases to verify vaccination status through immunisation card review, relying instead on the individual’s or caregiver’s verbal declaration. Similarly, verifying the age of the person interviewed was not possible in most cases. This can also be a source of misclassification between target or non-target population especially for children around 1 year old and adults around 30 years old, although the direction of any bias is difficult to predict. The proportion of children under 5 years was 18.5% in the interviewed sample, equal to the national estimate [21], suggesting little directional bias. Additional selection bias may have resulted from inaccurate population estimates in the cluster sampling frame: these were based on a 1998 census adjusted for estimated growth rates. All the above limitations might have biased VC estimates that we have used as a gold standard for classifying results of CLQAS simulations. Furthermore, these VC estimates were themselves subject to considerable imprecision. Our reference values for comparison of the CLQAS classification are thus imperfect, and weaken the strength of inference of our study.

Our CLQAS simulation also had limitations. We could only explore a 10 × 5 design due to the nature of our original survey dataset. It is known that higher sample sizes would achieve better accuracy of the CLQAS method, although, as discussed above, they would tend to negate its efficiency benefit over classical surveys. Furthermore, our original survey featured a sampling step of two between houses visited during the last stage of cluster selection; by contrast, CLQAS applications to date have used various sampling steps (nine or 18 for yellow fever in rural and urban areas respectively; three or six for polio [9]); because close proximity of households may increase the ICC, our findings may somewhat unfairly penalise the CLQAS method as it has been implemented. However, in practice our sampling step was such as to span nearly the full width of most villages in our sampling frame, which tended to be small. Furthermore, it is likely that most variability in VC is not within villages themselves, but at a higher administrative level, i.e. that differences in sampling steps may not greatly affect the ICC.

## Conclusions

This study suggests that small sample cluster surveys of 10 clusters x 15 individuals may be acceptably robust for practical applications of classifying VC at the local level. However, further studies are needed to establish the statistical robustness of these small samples in other settings. Based on this study, we do not recommend the CLQAS method as currently formulated for evaluating vaccination programmes.

## Competing interests

Authors declare no conflict of interest.

## Authors’ contributions

Conceived and designed the study: AM MRM ATa FF AT RFG FC. Performed the study: AM MRM FN MHK JS. Analyzed the data: AM MDM TR FC. Wrote the first draft of the manuscript: AM MRM FC. Contributed to the writing of the manuscript: all authors. Agree with manuscript results and conclusions: all authors. All authors read and approved the final manuscript.

## Funding Discloser

Th**i**s work was supported by Médecins Sans Frontières.

## Supplementary Material

Additional file 1 Cluster-level data for each health area. Click here for file
